# Variation of cerebrospinal fluid in specific regions regulates focality in transcranial direct current stimulation

**DOI:** 10.3389/fnhum.2022.952602

**Published:** 2022-09-02

**Authors:** Rajan Kashyap, Sagarika Bhattacharjee, Rose Dawn Bharath, Ganesan Venkatasubramanian, Kaviraja Udupa, Shahid Bashir, Kenichi Oishi, John E. Desmond, S. H. Annabel Chen, Cuntai Guan

**Affiliations:** ^1^Neuroimaging and Interventional Radiology, National Institute of Mental Health and Neurosciences (NIMHANS), Bengaluru, India; ^2^School of Computer Science and Engineering, Nanyang Technological University, Singapore, Singapore; ^3^Department of Neurophysiology, National Institute of Mental Health and Neurosciences, Bengaluru, India; ^4^Psychology, School of Social Sciences (SSS), Nanyang Technological University, Singapore, Singapore; ^5^InSTAR Program, Schizophrenia Clinic, Department of Psychiatry, National Institute of Mental Health and Neurosciences (NIMHANS), Bengaluru, India; ^6^Neuroscience Center, King Fahad Specialist Hospital Dammam, Dammam, Saudi Arabia; ^7^The Johns Hopkins University School of Medicine, Baltimore, MD, United States; ^8^Centre for Research and Development in Learning (CRADLE), Nanyang Technological University, Singapore, Singapore; ^9^Lee Kong Chian School of Medicine (LKC Medicine), Nanyang Technological University, Singapore, Singapore; ^10^National Institute of Education, Nanyang Technological University, Singapore, Singapore

**Keywords:** transcranial direct current stimulation (tDCS), realistic volumetric approach-based simulator for transcranial electric stimulation (ROAST), Systematic-Approach-for-tDCS-Analysis (SATA), current dose, brain volume, focality, age and sex

## Abstract

**Background:**

Conventionally, transcranial direct current stimulation (tDCS) aims to focalize the current reaching the target region-of-interest (ROI). The focality can be quantified by the dose-target-determination-index (DTDI). Despite having a uniform tDCS setup, some individuals receive focal stimulation (high DTDI) while others show reduced focality (“non-focal”). The volume of cerebrospinal fluid (CSF), gray matter (GM), and white matter (WM) underlying each ROI govern the tDCS current distribution inside the brain, thereby regulating focality.

**Aim:**

To determine the regional volume parameters that differentiate the focal and non-focal groups.

**Methods:**

T1-weighted images of the brain from 300 age-sex matched adults were divided into three equal groups- (a) Young (20 ≤ × < 40 years), (b) Middle (40 ≤ × < 60 years), and (c) Older (60 ≤ × < 80 years). For each group, inter and intra-hemispheric montages with electrodes at (1) F3 and right supraorbital region (F3-RSO), and (2) CP5 and Cz (CP5-Cz) were simulated, targeting the left- Dorsolateral Prefrontal Cortex (DLPFC) and -Inferior Parietal Lobule (IPL), respectively. Both montages were simulated for two current doses (1 and 2 mA). For each individual head simulated for a tDCS configuration (montage and dose), the current density at each region-of-interest (ROI) and their DTDI were calculated. The individuals were categorized into two groups- (1) Focal (DTDI ≥ 0.75), and (2) Non-focal (DTDI < 0.75). The regional volume of CSF, GM, and WM of all the ROIs was determined. For each tDCS configuration and ROI, three 3-way analysis of variance was performed considering- (i) GM, (ii) WM, and (iii) CSF as the dependent variable (DV). The age group, sex, and focality group were the between-subject factors. For a given ROI, if any of the 3 DV’s showed a significant main effect or interaction involving the focality group, then that ROI was classified as a “focal ROI.”

**Results:**

Regional CSF was the principal determinant of focality. For interhemispheric F3-RSO montage, interaction effect (*p* < 0.05) of age and focality was observed at Left Caudate Nucleus, with the focal group exhibiting higher CSF volume. The CSF volume of focal ROI correlated positively (*r ∼ 0.16, p* < 0.05) with the current density at the target ROI (DLPFC). For intrahemispheric CP5-Cz montage, a significant (*p* < 0.05) main effect was observed at the left pre- and post-central gyrus, with the focal group showing lower CSF volume. The CSF volume correlated negatively (*r ∼* –0.16, *p* < 0.05) with current density at left IPL. The results were consistent for both current doses.

**Conclusion:**

The CSF channels the flow of tDCS current between electrodes with focal ROIs acting like reservoirs of current. The position of focal ROI in the channel determines the stimulation intensity at the target ROI. For focal stimulation in interhemispheric F3-RSO, the proximity of focal ROI reserves the current density at the target ROI (DLPFC). In contrast, for intrahemispheric montage (CP5-Cz), the far-end location of focal ROI reduces the current density at the target (IPL).

## Highlights

- When the same tDCS setup is applied, some individuals receive focal stimulation while others have reduced focality.
- High volume of cerebrospinal fluid (CSF) in specific regions contributes to differences between focal and non-focal group.
- The location of CSF pockets in the brain relative to the placement of tDCS electrodes on the scalp influences the focality of tDCS current in the target region.
- CSF pockets that are in the path between target and reference electrodes, and that are close to the target, tend to direct current into the target region, so individuals with greater amounts of CSF in those pockets show greater tDCS focality in the target region.
- In contrast, CSF pockets that are closer to the reference electrode and farther from the target will flux most of the current towards the distant reference electrode, so individuals with greater amounts of CSF in those pockets show reduced tDCS focality in the target region.
- We suggest our computational modeling approach for the determination of tDCS focality to be used in healthy individuals and recommend future studies to extend it in patients with stroke or neurodegeneration who exhibit various locations and extent of CSF pockets.

## Introduction

Transcranial Direct Current Stimulation (tDCS) is an increasingly popular non-invasive brain stimulation technique used to treat neurological and psychiatric disorders (Filmer et al., 2014; Antal et al., 2017; Razza et al., 2020). In a conventional tDCS montage, anode and cathode are placed over the scalp, and low intensity of direct electric current (typically 1–2 mA) is injected to stimulate the targeted region of interest (ROI) in the brain (Nitsche and Paulus, 2000; Nitsche et al., 2007). The injected electric current that percolates the scalp to reach the brain induces an electric field that causes alterations in neurophysiology and behavior (Kim et al., 2014; Cabral-Calderin et al., 2016; Antonenko et al., 2019; Jamil et al., 2020). Studies have found that only 45% (approximately) of the injected current gets delivered to the brain, and the majority gets dissipated by the skull (Burger and van Milaan, 1943; Rush and Driscoll, 1968). The portion that reaches the brain gets distributed across the ROIs by the high conductive (∼1.6–1.8 S/m) cerebrospinal fluid (CSF) and by low conductive (∼ 0.12–0.30 S/m) white matter (WM) and gray matter (GM) (Burger and van Milaan, 1943; Geddes and Baker, 1967; Baumann et al., 1997; Haueisen et al., 1997; Akhtari et al., 2002; Nadeem et al., 2003). There are several ROIs in the brain and each ROI compartmentalizes a certain volume of CSF, GM, and WM. Naturally, the amount of CSF, GM, and WM in each ROI is crucial in steering the current across an individual’s brain (Datta et al., 2009). For example, a wide pocket of CSF will redirect more current, leading to a higher electric field in the region (Datta et al., 2009). However, our knowledge about the regional volumetric influence on the intensity of stimulation at target ROI and the factors (e.g., age, sex, tDCS electrode placement, and current dose) that may govern are limited.

In this aspect, there is a general desire to focalise the tDCS stimulation by maximizing the electric field strength at the target ROI. The variation in the amount of current reaching the target ROI (even when the tDCS set-up is the same for each person) is considered to be the source of inconsistency in the output of the tDCS (Chhatbar et al., 2017; Kirton et al., 2017; Vöröslakos et al., 2018; Caulfield et al., 2020b). In our previous work, we introduced Dose-Target-Determination-Index (DTDI), a simple estimate to quantify the focality of stimulation based on the current density (a measure of electric field strength) received at the target ROI and intermediary regions (for details, see methods) (Kashyap et al., 2021). DTDI ranges from 0 to 1, and values more than 0.75 indicate high focality (see “Materials and methods”) since it ensures that the targeted ROI of the brain is well-stimulated (Kashyap et al., 2021). To measure DTDI, we provide the computational toolbox Individual-Systematic-Approach-for-tDCS-Analysis (i-SATA) (Kashyap et al., 2021) that estimates the current density in 116 ROIs [parcellated using the automated Anatomical labeling (AAL) atlas (Tzourio-Mazoyer et al., 2002)] after a tDCS montage is simulated on an individual’s T1-weighted magnetic resonance image (MRI) of the brain that is segmented using realistic volumetric approach-based simulator for transcranial electric stimulation (ROAST, version 3.0) toolbox (Huang et al., 2019). The DTDI varies across individuals despite the tDCS montage specifications being fixed. The distribution of electric current in some individuals is focal (DTDI ≥ 0.75), whereas in others there is reduced focality. In this study, the subjects whose DTDI values are less than 0.75 will be considered as ‘non-focal.’ The primary aim of this study will be to determine the anatomical factors that contribute to the difference between the focal and non-focal groups.

Recently, a few studies have investigated the effect of global brain parameters (e.g., total CSF volume, total head volume, etc.) on the distribution of tDCS electric field strength. Antonenko et al. (2021) performed a study to investigate the relationship between electric field strength and the parameters of global head anatomy (total head-, CSF-, skull-, and skin- volume) in younger (20–35 years) and older (64–79 years) adults (*n* = 40). They observed an interaction between age group and total CSF volume, indicating strong linear associations in young compared to older adults. Bhattacharjee et al. (2022) investigated the effect of 16 global brain anatomical parameters (Total CSF-, GM-, WM- volume, brain torque, and dimensions) on electric field strength at the target ROI [dorsolateral prefrontal cortex (DLPFC) and inferior parietal lobule (IPL)] in young, middle-aged and older individuals (*n* = 240, 18–87 years of age) across both sexes. They tested two montages (targeting frontal and parietal regions) and found the total volume of CSF and GM to significantly associate with the amount of current reaching the target ROIs, though there are contributions from specific age-, sex-, and montage- dependent factors. Similarly, in our previous study, we also divided the samples into three age groups and two sexes and found focality to be lower in males starting only from middle age (40–60 years). We recommended a higher current dose to be used in tDCS studies recruiting individuals in that (and above) age range (Kashyap et al., 2021). Indahlastari et al. (2020) also simulated two montages (targeting frontal and motor regions) on 587 healthy adults (51–95 years) and found the current intensity at the target ROIs to be mediated by the global brain-to-CSF ratio, which decreases with increase in age. Another study in 2019 (Thomas et al., 2019) simulated the brain MRIs of five males and five females (27–47 years) with tDCS electrodes positioned at the left motor cortex and contralateral supraorbital (C3-SO). They found females to have higher current density than males owing to anatomical differences in the total volume of WM and GM. An interesting point observed in all these studies (Antonenko et al., 2019; Indahlastari et al., 2020; Kashyap et al., 2021; Bhattacharjee et al., 2022) is that population sample is subdivided into groups based on age and sex, and simulations are titrated by changing the tDCS parameters (montage position and current dose). This approach is robust and serves an additional purpose. In several tDCS-based experimental settings, it is difficult to obtain the MRIs of the subject. Therefore, these investigations based on demographic features (age and sex) can help researchers choose the tDCS parameters based on typical anatomical factors found in the experimental groups. Importantly, although these prior studies provided substantial evidence for age- and sex-related features of global brain morphometry that could influence the precision of tDCS, little is known about the contribution of the parameters of regional segmented anatomy (regional volume of CSF, WM, and GM) to the tDCS current distribution in the brain.

In this study, we investigated the parameters of regional segmented anatomy obtained from 116 regions (AAL parcellated) of the brain that contributed to the focality differences between the two groups (focal and non-focal). For this investigation, we simulated 300 T1-weighted brain MRIs of individuals (20–80 years of age) divided equally into three age-sex matched groups (Young, Middle and Older) across two montage positions [electrodes at- left frontal and right supraorbital (F3-RSO), and left temporo-parietal (CP5-Cz)] and two current doses (1 and 2 mA). The montages were chosen based on their translational utility and the placement of electrodes on the scalp (inter- and intra-hemispheric, see methods). For each montage, we measured the DTDI and categorized the individuals as either focal (DTDI ≥ 0.75) or non-focal (DTDI < 0.75). We calculated the regional CSF, GM, and WM volume of the 116 AAL parcellated brain regions using the computational anatomy toolbox (CAT) (Gaser and Dahnke, 2016). Finally, we evaluated the focality-based differences in regional anatomy by performing separate three-way factorial analysis of variance (ANOVA) on the CSF, GM and WM volumes of each ROI. The age cut-off points of the present sample were kept same as in our previous study (Kashyap et al., 2021) to have a consistency in the findings. Although the threshold of DTDI at 0.75 was inspired from previous nerve stimulation studies that found this threshold to be optimal in stimulating the target region (Tigra et al., 2016; Kosta et al., 2019), additional analysis was performed here to determine the optimality of DTDI (please see methods section “Additional analysis of focality”).

## Materials and methods

### Overview

We simulated 300 brain MRIs derived from 3 age groups (young, middle, and older) across 2 sexes (male and female) for the two montage positions (F3-RSO and CP5-Cz) and two current doses (1 and 2 mA). For each simulated head model, the DTDI was calculated as the measure of focality and thresholded at 0.75 to segregate individuals into two groups (focal and non-focal). The volume of CSF, WM, and GM from 116 brain regions was obtained for each MRI. Finally, the association of focality with regional volume and its dependence on age and sex were evaluated. A similar analysis was performed on the scores of 4 behavioral measures (cognition, anxiety, depression, and sleep) to investigate the phenotypic signature of focality.

### Brain data

The dataset used in the study was taken from publicly available Cambridge Centre for Aging and Neuroscience (Cam-CAN) inventory (Shafto et al., 2014; Taylor et al., 2017).^1^ The Cam-CAN project uses epidemiological, cognitive, and neuroimaging data to understand the aging brain. The repository provides a subset of data from 700 English-speaking healthy individuals (age range 18–88 years) with mini-mental state examination (MMSE) score above 27, having normal hearing and vision, and free from neurological or psychiatric conditions. We selected the T1-weighted brain MRI of 300 right-handed individuals (150 male) such that the images can be divided into three (uniformly spaced) age groups with 100 age-sex matched individuals (50 males and females) in each group comprising of—(a) Young adults (20 ≤ × < 40 years), (b) Middle adults (40 ≤ × < 60 years), and (c) Older adults (60 ≤ × < 80 years). The age range selected in the study was adopted from our previous work (Kashyap et al., 2021) as we observed focality to change significantly in males from middle age onwards. Consequently, in this work, we intended to explore the volumetric changes in the brain anatomy that are innate to each group and associate with the focality-based changes. The MRIs were collected from a 3T Siemens TIM Trio scanner with a 32-channel head coil using MPRAGE sequence (TR = 2,250 ms, TE = 2.99 ms, flip angle = 9^°^, Voxel size = 1 × 1 × 1 mm^3^, FOV = 256 × 240 × 192 mm^3^, GRAPPA: 2, TI: 900 ms).

### Simulation of transcranial direct current stimulation

The tDCS electrode placement followed the 10–20 electroencephalogram (EEG) labeling system to convey the position of anode and cathode over the scalp. Two montages with electrodes (Anode-Cathode) positioned at F3-RSO and CP5-Cz were simulated using ROAST (version 3.0) toolbox (Huang et al., 2019). For each MRI, the montages were simulated for two current doses (1 and 2 mA). Altogether, four tDCS configurations—(1) F3-RSO for 1 mA, (2) F3-RSO for 2 mA, (3) CP5-Cz for 1 mA, and (4) CP5-Cz for 2 mA were simulated across 3 age-groups with equal males and females. For all configurations, the electrode sizes were kept constant at 5 × 5 cm^2^ and the conductivity values for the various brain tissues were set at default [as mentioned in ROAST (Huang et al., 2019)] with WM (default 0.126 S/m); GM (default 0.276 S/m); CSF (default 1.65 S/m); bone (default 0.01 S/m); skin (default 0.465 S/m); air (default 2.5×10^−14^ S/m); gel (default 0.3 S/m); electrode (default 5.9×10^7^ S/m) (Burger and van Milaan, 1943; Geddes and Baker, 1967; Baumann et al., 1997; Haueisen et al., 1997; Akhtari et al., 2002; Nadeem et al., 2003). The ROAST segments the brain MRI and provides the simulated current density distribution for the virtual tDCS electrodes placed over the scalp. The simulated distribution was found to correlate with in-vivo electrophysiological measurements (Huang et al., 2017). The ROAST outputs were the location [x, y, and z coordinates (in mm)] of each brain area in the native space and the magnitude of current density (mA/m^2^) values corresponding to the location. In total, four tDCS configurations (2 montage positions × 2 current doses) applied on 300 individuals produced 1,200 simulations (300 individuals × 4 tDCS configurations) with target ROI at DLPFC for montage F3-RSO, and at IPL for montage CP5-Cz.

Although there are many possible combinations of tDCS montages and currents that could have been used for the simulations in the present investigation, the primary reasons behind our choice for the 4 tDCS configurations (2 montage positions and 2 current doses) that we used are- (1) *Encompassment of inter- and intra-hemispheric configurations*- In tDCS, the current travels between anode and cathode *via* the conductive brain tissue (Burger and van Milaan, 1943; Geddes and Baker, 1967; Akhtari et al., 2002; Nadeem et al., 2003). For the montage F3-RSO, the electrodes are placed across the two hemispheres of the brain. In this case, the current must percolate through the subcortical structures to reach the opposite hemisphere (Miranda et al., 2006; Bikson et al., 2010; Moliadze et al., 2010). In contrast, the montage CP5-Cz is on the same hemisphere, and the current most likely flow superficially (i.e., *via* the surface of the cortex). We anticipated that the different flow of current in the two montages will manifest discrete electric field patterns. Thus, the two montages can provide a representative understanding of the influence of regional volumetric parameters on focality of tDCS. (2) *Utility of the two montage positions*- Numerous studies have recommended the montage F3-RSO for the treatment of depression (Ammann et al., 2017; Moffa et al., 2020; Razza et al., 2020) and working memory (Nikolin et al., 2018). Similarly, CP5-Cz montage that stimulated the left IPL was used to modulate the reading behavior (Bhattacharjee et al., 2019a,b, 2020, 2022). Therefore, exploring focality with the two montage positions can have clinical importance. (3) *Benefits of the two current doses*- tDCS is considered safe for current doses up to 2 mA, and the vast majority of studies use intensity of either 1 or 2 mA (Jamil et al., 2017; Reinhart et al., 2017; Thair et al., 2017). Hence, the montages simulated for two widely practiced current doses will have broad applicability.

### Estimation of dose-target-determination-index

The i-SATA converts the coordinates of the brain from native space (obtained as the output of ROAST) to the standard space. There are two versions of i-SATA- (1) i-SATA (Talairach) (Kashyap et al., 2020) that converts to the Talairach brain space using the Talairach client (Lancaster et al., 2000), and (2) i-SATA (MNI) (Kashyap et al., 2021) that converts to Montreal neurological institute (MNI) space (Collins et al., 1994; Amunts and Zilles, 2001) using the 116 region-based AAL atlas from SPM anatomy toolbox (Eickhoff et al., 2005). In this work, i-SATA (MNI) was utilized to demarcate the anatomical boundaries of the 116 ROIs and estimate their current density. The magnitudes of current density received by the coordinates of an ROI were averaged to represent the current density of the region. DTDI is the ratio of the current density value at the target ROI to the peak current density value formed at any intermediary region.

$$D\,T\,D\,I=\frac{C\,u\,r\,r\,e\,n\,t\,d\,e\,n\,s\,i\,t\,y\,a\,t\,t\,h\,e\,T\,a\,r\,g\,e\,t\,R\,O\,I}{P\,e\,a\,k\,v\,a\,l\,u\,e\,o\,f\,c\,u\,r\,r\,e\,n\,t\,d\,e\,n\,s\,i\,t\,y\,f\,o\,r\,m\,e\,d\,a\,t\,a\,n\,y\,R\,O\,I}$$

The DTDI ranges from 0 (when no current reaches the Target ROI) to 1 (when the peak is formed at the target ROI), and values above 0.75 ensure that a considerable amount of current is reaching the target ROI.

For the montage F3-RSO with two current intensities (1 and 2 mA), the DTDI and the current density at the left DLPFC (target ROI) were obtained. Similarly, for the montage CP5-Cz and the two current doses, DTDI and current density were calculated for left IPL. For any tDCS configuration, two groups were delineated based on the DTDI value- (1) Focal group- which constituted individuals whose DTDI ≥ 0.75, and (2) Non-focal group- which represented individuals with DTDI < 0.75.

### Estimation of cerebrospinal fluid, white matter and gray matter of region-of-interests

The 300 MRIs were preprocessed in CAT (version 12.8) using the pipeline (with default parameters) inbuilt for the region-based morphometric analysis (Gaser and Dahnke, 2016). In this pipeline, the regional tissue volumes for an ROI defined in an atlas brain were obtained after mapping it to the individual brain using high dimensional spatial registration. The images were corrected for bias–field inhomogeneity, spatially normalized, and smoothened by an isotropic Gaussian kernel of 6 mm full width at half maximum. The partial volume estimation (Tohka et al., 2004) technique was used to segment the MRI computed the regional volume. This method considered every voxel in the image to be a mixture of three tissues, namely the GM, WM, and CSF. The fraction (p1, p2, and p3) of GM, WM, and CSF in each voxel was calculated such that p1 + p2 + p3 = 1. This was followed by the volumetric estimation of the ROIs in the native space. The 116 region-based AAL atlas (Tzourio-Mazoyer et al., 2002) was then warped from the standard MNI space to the native space using the inverse deformation field derived for each individual. The regional volume of GM, WM and CSF was then estimated after the fractional value of each voxel was multiplied by the voxel size. For example, the GM volume for an AAL demarcated ROI is the sum of p1 values contained within that ROI multiplied by the voxel size (similarly for WM and CSF volumes). The CSF-filled region can have a small amount of WM or GM volume (and vice-versa) owing to the partial volume effect. Typically, the GM/WM volumes for CSF regions are quite small compared to their corresponding GM/WM volumes. For instance, the CSF-filled ventricles can have very small values near the edges that account for the GM volume of that ROI. The regional volumes are in milliliters (ml) but were scaled by the total intracranial volume (Total volume of WM + GM + CSF) to correct for the variation in the brain size (Cousijn et al., 2012). Initially, age and sex were not regressed out since the association of these two factors with the volumetric parameters were of interest in the ANOVA analysis of the two groups (focal and non-focal, for details refer to the section- *Statistical Analysis of variation of focality*). However, studies have reported that age and sex can influence the volumetric results (Cousijn et al., 2012). Therefore, ANOVA analysis was repeated after regressing out age and sex from the volumetric parameters.

### Behavioral data

Much research has been devoted to the exploration of behaviors that are modulated by tDCS. A majority of the studies have found convincing evidence of the ability of tDCS to modulate cognition, anxiety, depression, and sleep (Ammann et al., 2017; Thair et al., 2017; Dondé et al., 2018; Moffa et al., 2020; Razza et al., 2020; Yamada and Sumiyoshi, 2021). Interestingly, the Cam-CAN repository provides scores for these behavioral measures- (1) MMSE for cognition, (2) hospital anxiety and depression scale (HADS) for anxiety (HADS_anxiety), (3) HADS for depression (HADS_depression), and (4) sleep quality (Pittsburg sleep quality index, PSQI) for the 300 individuals considered in the study. This motivated us to explore whether any of these behavioral measures exhibited significant differences between the focal and non-focal groups. Investigating the phenotypic trend expressed in focality can be useful when an MRI of a subject is not available (or cannot be done), and there is an intention to identify if the subject would receive focal stimulation for the chosen tDCS configuration.

### Statistical analysis of variation of focality

For each tDCS configuration, the individuals from three age groups (Young, Middle, and Older) across both sexes (males and females) were segregated into the focal and non-focal groups. For each individual, the regional CSF, GM and WM volumes were extracted from 116 ROIs. For each montage (2 of them), for each tDCS current value (2 of them), and for each ROI (116 of them), we performed three 3-way ANOVAs separately, one with GM as the dependent variable (DV), one with WM as the DV, and one with CSF as the DV. Each of the ANOVAs had between-subject factors such as age group, sex, and focality group as independent variables. For a given ROI, if any of the 3 DV’s showed a significant (*p* < 0.05, *Bonferroni corrected*) main effect or interaction involving the focality group, then that ROI was to be considered as a “focal ROI.” For each group, the Shapiro-Wilk test was performed to verify the assumptions of normality, and for each ANOVA, Mauchly’s test evaluated the assumption of sphericity. *Post hoc* analysis was performed to further characterize the main and interaction effects of focality. Finally, the current densities at the target ROI were correlated (using Pearson correlation) with the volumetric values of focal ROI. To check whether the correlation has an issue of heteroscedasticity, we used the White test. Additionally, for each tDCS configuration (montage and current dose), we also correlated the CSF volume of the focal ROI with the current density values of the target ROI for the focal and the non-focal group separately. If the correlation coefficient is significantly (*p* < 0.05) different between the two groups, then it will indicate that the focality of stimulation is influenced by the regional anatomy of the brain. Finally, we performed ANOVA analysis on the four behavioral measures (MMSE, HADS_anxiety, HADS_depression, PSQI).

### Additional analysis of focality

The DTDI quantified focality in tDCS and was well integrated with the i-SATA (MNI) toolbox (Kashyap et al., 2021). This metric was inspired by a similar measure widely followed in nerve stimulation techniques where the targeted region is considered to be adequately stimulated for values above the threshold of 0.75 (Deurloo et al., 2000; Tigra et al., 2016; Kosta et al., 2019). Since such a high threshold ensures that the target ROI will be effectively stimulated, we followed the same value (0.75) as the threshold of DTDI to segregate the focal and non-focal groups. We understand that the threshold plays a vital role, and there is a need to identify the limit until which our findings may remain valid. To examine this, we took an iterative approach wherein we reduced the threshold of DTDI in steps of 0.01 (from the previous threshold) and repeated the 3-way ANOVA analysis (for the volume parameter). The threshold at which null/insignificant findings appeared, the iteration stops. For example, in the first iteration, we separated the focal and non-focal groups for a threshold of 0.74 (i.e., 0.75–0.01) and performed the ANOVA analysis. We checked the consistency of the results of ANOVA obtained for the DTDI threshold of 0.74 with the findings obtained previously with a threshold of 0.75. If the results were consistent (i.e., *p*-values are significant), then in the second iteration, we repeated the previous steps for the threshold of 0.73 (i.e., 0.74–0.01). Otherwise, we stopped iterating further.

## Results

### Overview

The focus of the study was to investigate the contribution of regional volume to differences in focality across the two montages and two current intensities. Interestingly, the results were driven by montage position and current intensities do not alter them. For the F3-RSO configurations and two current intensities, the CSF volume at the left caudate nucleus was significantly higher in the focal group (for the older group only). The CSF volume correlated positively with the current density at the left DLPFC. For the configurations with electrode position at CP5-Cz and two current intensities, a higher CSF volume was observed for the non-focal group at the left pre- and post-central gyrus. The correlation between the CSF volume and the current density at the left IPL was negative. Surprisingly, the results from the two montages (F3-RSO and CP5-Cz) appeared to contradict each other.

### Output of montage F3-right supraorbital region for 1 and 2 mA

**FIGURE 1:**
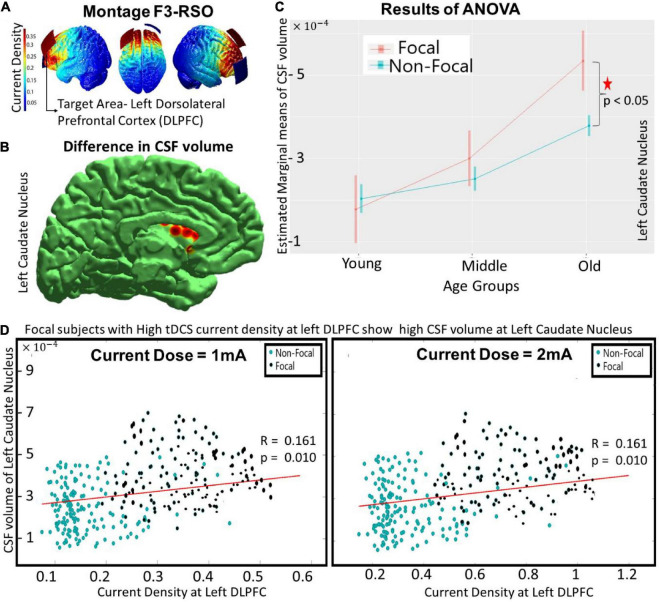
The differences between focal and non-focal groups for the montage F3-RSO (at 1 mA) with—**(A)** showing the spread of current density over an individual’s brain with target ROI at left DLPFC, **(B)** reflecting the location of “focal” ROI at left caudate nucleus wherein significant (*p* < 0.05) differences in CSF volume was observed between focal and non-focal groups, **(C)** highlighting that the difference was predominantly in the older group with focals having higher CSF volume, and **(D)** indicating that the CSF volume at focal ROI (left caudate nucleus) correlated (positively) with the current density at the target area (left DLPFC). The results remained similar as the current intensity was increased to 2 mA (as shown in **D**).

The key motive of the study was to comprehend focality and its interaction with age and sex. To this, the current densities were simulated for all individuals. As an example, we show (Figure 1A) the spread of the simulated current density over an individual’s brain along with the montage electrodes placed at F3 and RSO. The DTDI of all individuals were estimated for the target area at left DLPFC. The individuals were divided into focal and non-focal groups based on the DTDI threshold prefixed at 0.75. Table 1 shows the range (Mean ± Std) of DTDI for the two groups (focal and non-focal) across the two tDCS configurations (1 and 2 mA). The number of males and females in each group (focal/non-focal) and their distribution across young, middle and older age groups were provided. All groups were normally distributed (*W* > 0.97, *p* > 0.05), and Mauchly’s test also indicated that the assumption of sphericity was not being violated (*p* > 0.05). For the four groups across two configurations, Table 1 lists the scores (Mean ± STD) of the four behavioral measures for the one-way ANOVA analysis. We did not find a significant (*p <* 0.05) difference between the groups (focal and non-focal) for the behaviors. These behavioral measures were included to evaluate if there are any association of focality with behavioral scores. A significant association could help the tDCS community to select subjects (based on that behavioral score) for whom the applied tDCS will optimally stimulate the target ROI. Our lack of significant differences in anxiety and depression scores for focal and non-focal groups could be due to the fact that our subjects were obtained from a database of healthy individuals. Future investigations could examine the relationship between such behavioral measures and focality in patient populations.

**TABLE 1:** Enlists the composition of the focal and non-focal group for the montage F3-RSO and the two current doses (1 and 2 mA).

Variable name		Current dose = 1 mA		Current dose = 2 mA	
		Focal (*n* = 138)	Non-focal (*n* = 162)	Focal (*n* = 144)	Non-focal (*n* = 156)
Focality	DTDI (mean ± STD)	0.87 ± 0.11	0.56 ± 0.18	0.89 ± 0.08	0.57 ± 0.14
Sex	Total male	64	86	74	76
	Total female	74	76	70	80
Age-group	Total young	53	47	55	45
	Total middle	44	56	46	54
	Total older	41	59	43	57
Behavioral measures	MMSE	29.1 ± 1.21	28.8 ± 1.23	29.0 ± 1.21	28.8 ± 1.23
	HADS_anxiety	5.1 ± 3.16	4.9 ± 3.58	5.0 ± 3.35	5.0 ± 3.42
	HADS_depression	2.97 ± 3.09	2.77 ± 2.83	3.06 ± 3.04	2.97 ± 2.57
	PSQI	5.22 ± 3.96	5.46 ± 3.50	5.34 ± 3.72	5.47 ± 3.73

For each group, the number of—(i) males and females, and (ii) young, middle and older agers are provided along with their variation (Mean ± STD) in—(i) DTDI, and (ii) four behavioral measures.

**FIGURE 2:**
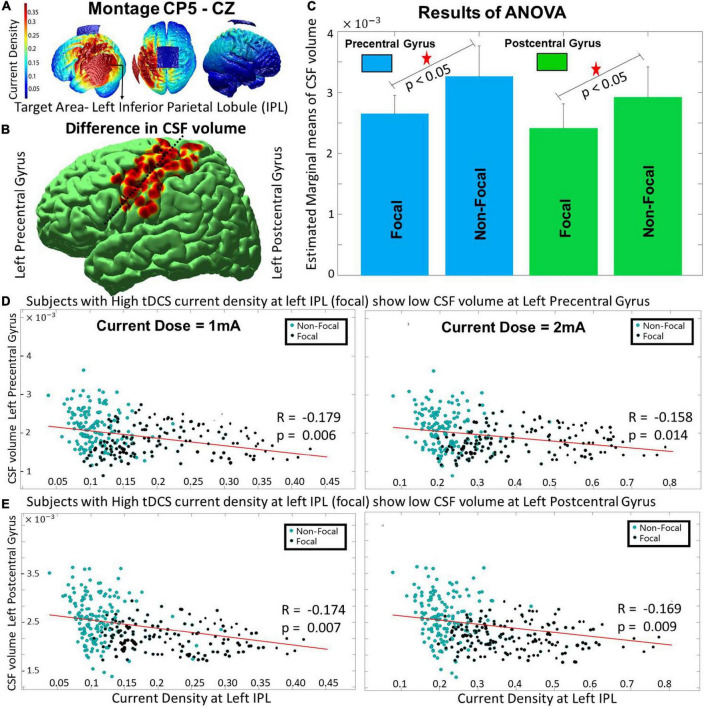
The differences between the focal and non-focal group for the montage CP5-Cz with- **(A)** showing the spread of current density on an individual’s brain with target ROI at left IPL, **(B)** reflecting the location of focal ROIs- left pre- and post-central gyrus (nearby locations separated by dashed line) wherein significant (*p* < 0.05) differences in CSF volume were observed, **(C)** highlighting that the focal group had lower CSF volume at the focal ROIs, **(D,E)** indicating that the decrease in CSF volume at focal ROIs (pre- and post-central gyrus) is linked with an increase in current density at the target area (left IPL). The results for the current dose of 2 mA also showed a similar trend.

The focal ROIs were determined for all the regional volume parameters (CSF, GM and WM). A three-way ANOVA (performed on the groups obtained from montage F3-RSO at 1 mA) revealed significant differences (*p* < 0.05, *Bonferroni corrected*) in the regional CSF volume at the left caudate nucleus. There was a significant main effect of age [*F* (1, 2) = 85.84, *p <* 10^−14^] and an interaction effect of age and focality [*F* (1, 2) = 16.38, *p < 0.0001*] (Shown in red in Figure 1C). The *post hoc* comparison showed that the difference was significant (*p* < 0.0001) for the older group only, with subjects in the focal group showing higher volume (Figure 1C) than the non-focal group. The results remained similar for the tDCS configuration F3-RSO at 2 mA (not shown). The correlation between the current density at the left DLPFC (Target ROI) and the CSF volume at left caudate nucleus (focal ROI) was positive (∼ 0.16) and significant (*p* < 0.01) for both configurations (Figure 1D- 1 and 2 mA). The results of the White test (*p* = 0.12) confirmed that there is no heteroscedasticity. For both current doses (1 and 2 mA), the correlation of current density of left DLPFC with CSF volume of left caudate nucleus are calculated for the (i) Focal Group (*r* ∼ 0.28, *p* < 0.01) and (ii) non-focal (*r* ∼ 0.09, *p* > 0.05). A significant (*p* < 0.05) difference in the correlation coefficient between the two groups exists. The focal group with high current density at left DLPFC showed high CSF volume at left caudate nucleus.

Similar to previous studies (Gur et al., 1991; Lemaître et al., 2005; Mortamet et al., 2005; Lotze et al., 2019; Kijonka et al., 2020), significant effects of age and sex were observed in our analyses. Briefly, the significant (*p* < 0.05, Bonferroni corrected) main effect of age for the—(1) CSF volume was found in 66 ROIs (distributed throughout the brain), (2) WM was found in 9 ROIs (mainly in subcortical structures), and (3) GM was found in 11 ROIs (cortical and subcortical). The main effect of sex was found for the CSF volume only across the 4 ROIs (frontal and parietal areas). The other interaction effects across the combinations (age and sex, sex and focality, and age, sex and focality) were insignificant for the three regional volume parameters across the ROIs. Although significant effects of age and sex were observed in our analyses, we do not present the details of these effects here because age and sex had been investigated extensively in previous studies, and we do not wish to divert the attention from focality. However, as noted above, there was an age-focality interaction in the left caudate nucleus. We also performed a similar analysis after regressing out age and sex from the volumetric parameters. This did not alter the existing results, however, we found a significant [*F*(1, 2) = 72.34, *p <* 10^−12^) effect of age and an interaction effect of age and focality [*F*(1, 2) = 12.64, *p* < 0.001] in the regional CSF volume for the right caudate nucleus. The *post hoc* comparison also showed that the difference was only significant (*p* < 0.0001) for the older group. However, the correlation of the CSF volume at the right caudate nucleus with the current density at the target area (left DLPFC) was insignificant for both current doses (figure not shown).

### Output of montage CP5-Cz for 1 and 2 mA

The spread of the current density over an individual’s brain is shown in Figure 2A for the montage positioned at CP5-Cz with the target ROI at left IPL. Similar to the F3-RSO montage, the focal and non-focal group composition for the CP5-Cz configurations (1 and 2 mA) is in Table 2. The table shows the mean and standard deviation of DTDI for the two groups (focal and non-focal) across the two tDCS configurations (1 and 2 mA). The number of males and females in each group (focal/non-focal) is provided. The individuals in each group were distributed across young, middle, and older ages. The groups were normally distributed (*W >* 0.97, *p* > 0.05) and assumption of sphericity was not violated (*p* > 0.05). The scores for the four behaviors (MMSE, HADS_anxiety, HADS_depression, and PSQI) are also enlisted (Mean ± STD). The one-way ANOVA analysis of the scores of 4 behavioral measures for the two groups (focal and non-focal) did not find a significant difference between them.

**TABLE 2:** Same as Table 1 for the montage location CP5-Cz with two current doses (1 and 2 mA).

Variable name		Current dose = 1 mA		Current dose = 2 mA	
		Focal (*n* = 126)	Non-focal (*n* = 174)	Focal (*n* = 135)	Non-focal (*n* = 165)
Focality	DTDI (mean ± STD)	0.87 ± 0.12	0.55 ± 0.15	0.89 ± 0.11	0.57 ± 0.14
Sex	Total male	59	91	64	86
	Total female	67	83	71	79
Age-group	Total young	54	46	54	46
	Total middle	36	64	41	59
	Total older	36	64	40	60
Behavioral measures	MMSE	29.1 ± 1.21	28.8 ± 1.23	29.0 ± 1.21	28.8 ± 1.23
	HADS_anxiety	5.1 ± 3.16	4.9 ± 3.58	5.0 ± 3.35	5.0 ± 3.42
	HADS_depression	2.97 ± 3.09	2.77 ± 2.83	3.06 ± 3.04	2.77 ± 2.77
	PSQI	5.18 ± 3.96	5.46 ± 3.50	5.34 ± 3.72	5.47 ± 3.73

Across the volume parameters, the two-way ANOVA analysis (performed on the groups obtained from 1 mA) revealed that the CSF volume at two focal ROIs, namely the left precentral [*F*(1, 1) = 10.15, *p <* 10^−5^] and postcentral gyrus [*F*(1, 1) = 18.76, *p <* 10^−7^] (shown with a black dotted line dividing the two red regions in Figure 2B) showed a significant main effect of focality (*p* < 0.05, *Bonferroni corrected*). The estimated marginal means found the non-focal group to have higher CSF volume compared to the focal group for both pre- and post-central gyrus (Figure 1C). The other volume parameters (WM or GM) were not significant. The result was also similar to the segregated groups for the current intensity of 2 mA (result not shown). A negative but significant (*p* < 0.01) correlation (*r*-value ranging from –0.15 to –0.18) was observed between the current density values at the target ROI (IPL) and the CSF volume of the focal ROIs (pre- and post-central gyrus) across the two current intensities (Figures 2D,E - 1 and 2 mA). The results of the White test (*p*-values ranging from 0.2306 to 0.2672) confirmed that there was no issue of heteroscedasticity in the correlation analysis across the two focal ROIs and two current doses. For both current doses (1 and 2 mA), the correlation of current density of IPL with CSF volume of pre- and post-central gyrus are calculated for the (i) Focal Group (*r* ∼ –0.33, *p* < 0.01) and (ii) non-focal (*r* ∼ –0.14, *p* < 0.01) groups. A significant (*p* < 0.05) difference in the correlation coefficient between the two groups exists. On a side note (as explained previously), the main effect of age and sex were similar for the two montages (CP5-Cz and F3-RSO). The interaction effects across the combinations (age and sex, age and focality, sex and focality, and age, sex and focality) were insignificant for all the volumetric parameters (regional CSF, WM, and GM).

Another interesting aspect that can be seen in both montages (F3-RSO and CP5-Cz, Figures 1, 2) across all the focal ROIs was that the distribution pattern of the current density does not appear to be the same for the two current doses (1 and 2 mA). This observation is in concordance with our previous findings, where we found a non-linear increase in current density with an increase in the current dose that is predominant in the older (> 60 years of age) population (Kashyap et al., 2021). This non-linearity has been reported in several other studies (Brunoni et al., 2012; Hsu et al., 2015; Habich et al., 2020) and might be the reason behind the observed differences in the pattern of distribution. This has been extensively discussed in our previous study (Kashyap et al., 2021); and we again report it here since the observation replicates.

### Additional analysis with varying dose-target-determination-index threshold

For a tDCS configuration, we reduced the DTDI threshold in steps of 0.01. For the tDCS configuration F3-RSO at 1 mA, findings that were obtained with the predefined threshold of 0.75 were obtained until we have reduced the threshold to 0.70. For the tDCS configuration F3-RSO at 2 mA, the DTDI threshold could only be reduced to 0.73. Similarly, for the montage CP5-Cz at 1 and 2 mA, the DTDI can be reduced to 0.70 and 0.72, respectively. Altogether, the four tDCS configurations suggest that the threshold of 0.75 can be an acceptable milestone to gauge tDCS focality.

## Discussion

The present work finds the CSF volume of certain brain regions (referred as *“focal ROIs”*) to contribute to the focality based differences in tDCS. The CSF with the highest conductivity of all brain tissues (Datta et al., 2009; Moliadze et al., 2010; Reinhart et al., 2017; Thair et al., 2017) and varying concentrations across the ROIs plays an essential role in channeling the percolated tDCS current. Higher concentration in a focal ROI drags more current toward it, molding the channel of current flow between the two electrodes (Holdefer et al., 2006; Datta et al., 2009). The location of this focal ROI was montage dependent and was a factor determining the difference between the focal and non-focal groups. We will discuss this for each montage in the next section.

For the interhemispheric placement of electrodes in the montage F3-RSO, the focal group exhibited high CSF volume at the left caudate nucleus (focal ROI). The correlation between CSF volume and the current density at the left DLPFC was positive (Figure 1D), signifying that the increase in CSF volume in the focal ROI was associated with the increase in current density at the target ROI. It is interesting to see that the current in a cortical area is regulated by the CSF volume of the subcortical region. We can possibly explain this phenomenon as the *Target ROI-Focal ROI continuum* of current in the brain. The CSF provides a channel for current from one electrode to percolate to the subcortex and reach the other hemisphere (Miranda et al., 2006; Bikson et al., 2010; Moliadze et al., 2010). The focal ROI (caudate nucleus) in the striatum falls within this pathway. The higher CSF volume in the focal ROI drags more current toward the target ROI minimizing the spread of the electric field. Since the target ROI is near the focal ROI, there is a continuum of current between them, leading to an increase in the current density (for the focal group). The focality gets further enhanced for the older group (see Figure 1C) since it is evident that the atrophy of the prefrontal cortex and the subcortical regions, including caudate nucleus accelerates after the age of 60 years (Raz et al., 2005; Fjell et al., 2009; Dima et al., 2022). The atrophied areas generate more space for CSF volume to fill it (Murphy et al., 1992; Pfefferbaum et al., 1994; Good et al., 2001), thereby creating provision for more current to get siphoned into the channel. This might also be associated with the release of neuromodulators such as dopamine, serotonin, and acetylcholine that mediate the effect of tDCS (Nitsche et al., 2006, 2009; Monte-Silva et al., 2010). Two recent studies that stimulated the left DLPFC (using a similar montage) found tDCS-induced dopamine release in the striatal areas (Fonteneau et al., 2018; Fukai et al., 2021). The CSF is involved in the distribution of neuromodulators across the brain (Taber and Hurley, 2014; Bjorefeldt et al., 2018) and it is possible that the higher CSF volume that reserves the tDCS current in striatum triggers the release of dopamine. Since adequate information regarding the relationship between CSF volume in the striatum and the amount of dopamine released due to tDCS is unknown, it is difficult to say how focality amalgamates in this coordination.

For the intra-hemispheric CP5-Cz electrode placement, two focal ROIs at pre- and post-central gyrus can be observed approximately under and adjacent to Cz electrode, where the current flow is expected to terminate. Interestingly, the two focal ROIs show higher CSF volume for the non-focal group. The two regions lying at the end of the channel cause the majority of injected current to be carried away to those areas. Possibly, they may also drain by shunting the current from adjoining areas (Bikson et al., 2010; Moliadze et al., 2010). The continuum of current flow is disrupted due to the far-off positioning of the focal ROIs from the target ROI leading to a decrease in current density at the left IPL (for the focal group).

At this point, it may appear that the observations of intra-hemispheric montage CP5-Cz contradict the findings of inter-hemispheric montage F3-RSO. For instance, the focal ROIs in CP5-Cz have lower CSF volume for the focal group, whereas the same group in F3-RSO exhibits higher CSF volume. We will explain the finding for both montages by looking at this as a “watershed” phenomenon. This analogy is inspired by the explanation made by Reinhart et al. (2017), wherein the tDCS current that percolates the skull is presumed to behave like water sharing the similar property of following the path of least resistance. According to the phenomena, the percolated tDCS current is like uphill rainwater, and the CSF volume of the brain areas (forming the current channel) is like the descending stream. The focal ROIs are the reservoirs (or clusters) of current situated in the channel between the two tDCS electrodes. Based on the principle that guides the continuity of current (Halliday et al., 2010), the nearer the target area is to the reservoir, the higher will be the flux of current and the resulting current density in the areas (as seen in the montage F3-RSO). Opposingly the clusters located at the end of channel (near the sink) will flux most of the current toward the distant reservoir, lowering the current density at the target ROI stationed at an intermediate position in the channel (as seen in the montage CP5-Cz). This is in support of the findings of Datta et al. (2009) where they found that the channels of high conductivity CSF perfusing the underlying cortex have clusters of high current density at distinct areas with wide pockets of CSF that influence the overall current flow in the brain due to tDCS. Our study sorted the nature with which montage placement interacts with such regional CSF pockets to steer the current at the target ROI.

## Limitations and future direction

The simulations approximate the electrical field generated inside an individual’s head, and there exist some constraints that limit the electric field modeling (Louviot et al., 2022). The two most concerning limitations that may impact the results of the study is the- (i) the use of fixed conductive value for the brain tissues in all individuals, and (ii) isotropy assumed in the layers of the brain tissues, especially in the deeper layers of the cortex (Aström et al., 2012). There exists another limitation from the analysis point of view since the two montages selected here (CP5-Cz and F3-RSO) are opposite in terms of configuration (inter- and intra-hemispheric) and target different brain regions. Therefore, it may be difficult to ascertain the contribution of the focal ROIs and/or the tDCS montage in the differences reported in the results. In the present study, the montages were adopted from previous computational and experimental studies that have shown them to be optimal in stimulating the target ROI (Bai et al., 2014; Ammann et al., 2017; Bhattacharjee et al., 2019a,b, 2020). However, to overcome the limitation, an ROI can be assumed a priori, a set of inter- and intra- hemispheric montages that appropriately target that ROI can be determined computationally and experimentally [as done in several studies (Bai et al., 2014; Bhattacharjee et al., 2019b)]. Then the anatomical factors that account for the differences between focal and non-focal group could be examined. Future simulation-based studies can mitigate these constraints to estimate the factors that regulate focality in tDCS.

In this paper we have used our DTDI measure as an index of “focality,” which is based on the distribution of current density across the ROIs of the brain. A maximum DTDI value of 1 indicates that the current density at the target ROI is maximum compared to other ROI’s. Another study delineated focality for transcranial magnetic stimulation (TMS) based on the volume and depth of the ROI (Deng et al., 2013). It will be interesting to incorporate the other measure of focality and determines its effectiveness in predicting the outcome of tDCS. Another concern that the study raises is the observed nonlinearity between current dose and density in the target ROI. Though the investigation is beyond the scope of the present study, future exploration of this phenomenon is needed.

Also, previous studies have fixed the stimulation parameters for all the individuals in a group, leading to inconsistency in the output of tDCS (Chhatbar et al., 2017; Thair et al., 2017; Caulfield et al., 2020a). Recent studies advocate individual-based tuning of parameters to increase the efficacy (Evans et al., 2020; Kashyap et al., 2020, 2021), raising the need to calibrate focality in tDCS from two perspectives. One such requirement is to gauge focality when multiple ROIs (or network of ROIs) need to be targeted (To et al., 2018). Another vital aspect is to decipher the interplay of local (Regional CSF, GM and WM) and global (Total CSF, GM and WM) parameters. Having said that, the other area where we can extend the study is—(1) In patients with a neurodegenerative or neurological disorder (e.g., stroke) wherein the CSF pockets get extended (due to atrophy), indicating the need to compare different tDCS configurations to see which one produces the best focality in an individual patient (Handiru et al., 2021), (2) by including several other widely used montages with the extra-cephalic placement of electrodes, (3) by using brain atlases with finer parcellations for qualitative mapping of the current densities with the regional anatomy, and (4) to identify a phenotypic biomarker of focality using datasets which include a wide spectrum of behavioral measures. Given the inter-individual variability of response (clinical symptoms, cognitive/behavioral measures) to tDCS, it will be also interesting to examine the utility of “focality-based” analyses (along the lines reported here) to explain the variation in extant datasets that have concurrent brain imaging data.

## Conclusion

In this work, we explored the behavior of regional CSF and its interaction with demographic factors in steering the current at the target ROI across the two kinds of montages. We find the high CSF volume of “focal” ROIs to govern focality depending on their position in the channel that is created by the passage of current between the tDCS electrodes. Focal ROIs that are in the path between target and reference electrodes (as seen in F3-RSO), and are close to the target, tend to direct current into the target region. Hence, individuals with greater amounts of CSF in those focal ROIs show greater tDCS focality in the target region. In contrast, focal ROIs closer to the reference electrode and farther from the target (as seen in CP5-Cz) will flux most of the current toward the distant reference electrode, so individuals with greater amounts of CSF in those focal ROIs will show reduced tDCS focality in the target region.

## Data availability statement

Publicly available datasets were analyzed in this study. The data is publicly available at https://camcan-archive.mrc-cbu.cam.ac.uk/dataaccess/.

## Ethics statement

The studies involving human participants were reviewed and approved by the Publicly available database (Cam-CAN). Cam-CAN study was conducted in compliance with the Helsinki Declaration, and was approved by the local ethics committee, Cambridgeshire 2 Research Ethics Committee (reference: 10/H0308/50). The ethics committee waived the requirement of written informed consent for participation.

## Author contributions

RK designed the overall study, performed the analysis, wrote the manuscript, and prepared the figures. SaB was involved in technical discussion, simulation analysis, and manuscript correction. RDB, GV, and KU provided vital inputs during the revision of the manuscript. ShB provided technical guidance. KO suggested improvements in technical design and was involved in manuscript correction. JD provided overall guidance to the work and was involved in technical tuning and in manuscript preparation. SC was involved in technical discussion and manuscript correction. CG obtained the funding for the work and was involved in technical discussion and monitoring. All authors contributed to the article and approved the submitted version.

## Footnotes

1
http://www.mrc-cbu.cam.ac.uk/datasets/camcan/
